# Particle-Filter-Based Fault Diagnosis for the Startup Process of an Open-Cycle Liquid-Propellant Rocket Engine

**DOI:** 10.3390/s24092798

**Published:** 2024-04-27

**Authors:** Jihyoung Cha, Sangho Ko, Soon-Young Park

**Affiliations:** 1 Centre for Aeronautics, Cranfield University, Cranfield MK43 0AL, UK; jihyoung.cha@cranfield.ac.uk; 2 Department of Smart Air Mobility, Korea Aerospace University, 76 Hanggongdaehang-ro, Deogyang-gu, Goyang-si 10540, Republic of Korea; 3 Rocket Engine Department, Korea Aerospace Research Institute, 169-84 Gwahak-ro, Daejeon 34133, Republic of Korea; psy@kari.re.kr

**Keywords:** fault detection and diagnosis, particle filter, CUSUM algorithm, multiple-model method, liquid-propellant rocket engine, startup process

## Abstract

This study introduces a fault diagnosis algorithm based on particle filtering for open-cycle liquid-propellant rocket engines (LPREs). The algorithm serves as a model-based method for the startup process, accounting for more than 30% of engine failures. Similar to the previous fault detection and diagnosis (FDD) algorithm for the startup process, the algorithm in this study is composed of a nonlinear filter to generate residuals, a residual analysis, and a multiple-model (MM) approach to detect and diagnose faults from the residuals. In contrast to the previous study, this study makes use of the modified cumulative sum (CUSUM) algorithm, widely used in change-detection monitoring, and a particle filter (PF), which is theoretically the most accurate nonlinear filter. The algorithm is confirmed numerically using the CUSUM and MM methods. Subsequently, the FDD algorithm is compared with an algorithm from a previous study using a Monte Carlo simulation. Through a comparative analysis of algorithmic performance, this study demonstrates that the current PF-based FDD algorithm outperforms the algorithm based on other nonlinear filters.

## 1. Introduction

Liquid-propellant rocket engines (LPREs) are mostly utilized for space launch vehicles (SLVs) or reusable launch vehicles (RLVs) for New Space because of their higher specific impulse and the accurate control capability required for precise injection into orbit [1,2,3,4,5]. However, given the complexity of LPREs and their multitude of components, achieving high reliability necessitates employing condition-based maintenance techniques. These include non-destructive inspection (NDI), fault detection and diagnosis (FDD), and strategies for prognosis and health management (PHM) based on sensor measurements obtained from the engines [6,7,8,9,10,11,12,13,14,15,16,17,18,19,20,21,22,23,24,25,26]. In terms of the FDD of LPREs, researchers have actively studied two distinguishing methods: data-driven and model-based methods. Due to the insufficiency of LPRE fault scenarios during actual firing tests, model-based methods are widely studied. Typically, these methods are based on the fault characteristics that are derived from a mathematical model with artificially inserted faults [14,15]. In the same context, this study focuses on developing a new FDD methodology using measurement data with these motivations and requirements.

**Figure 1:**
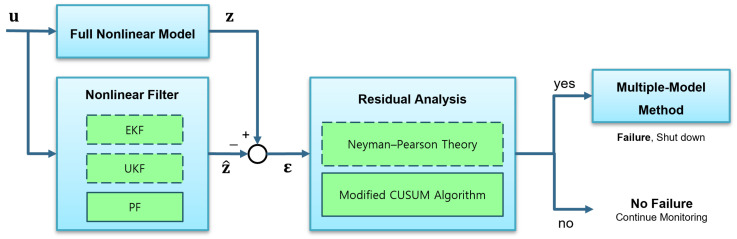
The structure of the FDD algorithm.

In the case of LPRE operational failures, Refs. [27,28] show that a significant proportion (exceeding 30%) of engine failures occur in the startup process due to various factors: leakage and blockage in the propellant pipeline, combustion instabilities caused by the water hammer in the priming process, inappropriate initial setting and opening times of each valve, etc. Most of these failures were immediately catastrophic, so the failures caused not only mission failure but also damage to both materials and individuals. To resolve these problems, in a previous study, Cha et al. [21] applied the extended Kalman filter (EKF) and unscented Kalman filter (UKF) as a model-based method to develop an FDD algorithm that could physically analyze fault conditions in the startup process of an LPRE. However, the usage of the two nonlinear Kalman filters has some limitations, namely, false alarms due to linearization, global approximation, and the assumption errors of the PDF of the fault condition [29,30] in the residual generation and fault detection processes with the Neyman–Pearson (NP) theory [31]. Therefore, in order to overcome these limitations of the previous study, this study proposes a new FDD algorithm employing a particle filter (PF) and a modified cumulative sum (CUSUM) algorithm, as shown in Figure 1. In the figure, the process of the FDD algorithm is delineated: the current study (depicted within solid boxes) and the previous study [21] (represented by dashed boxes).

To validate the performance of the proposed FDD algorithm, we employed two fault scenarios, a turbopump efficiency deficiency and pipeline blockage, which are common sources of LPRE failures [27]. The dynamic response data were acquired by applying fault scenarios to the mathematical model of the LPRE described in Ref. [15]. Then, we designed the filters, generated residuals, and detected and diagnosed each fault with the CUSUM algorithm and multiple-model (MM) method using the residuals. For each process of the FDD algorithm, a Monte Carlo simulation was conducted to compare the performance of each FDD algorithm. This work analyzed and compared the FDD algorithms quantitatively and qualitatively under random noise conditions. Through this work, we demonstrate that the FDD algorithm with the PF and CUSUM algorithm has superior performance compared to those in the previous study.

In this study, we selected an open-cycle LPRE as a base model to study, and a brief introduction is presented in Section 2. Section 3 explains the PF to generate residuals, which are the basis of decision-making in the FDD algorithm, and Section 4 describes the FDD algorithm, which is composed of a modified CUSUM algorithm to detect a fault and the MM method to diagnose the fault using the residuals. Then, Section 5 compares the results of the newly proposed FDD algorithm with those of the previous study using the EKF/UKF. Finally, in Section 6, the concluding remarks and limitations of this study are discussed.

## 2. Dynamics of LPREs

### 2.1. Dynamic Simulation of LPREs

**Figure 2:**
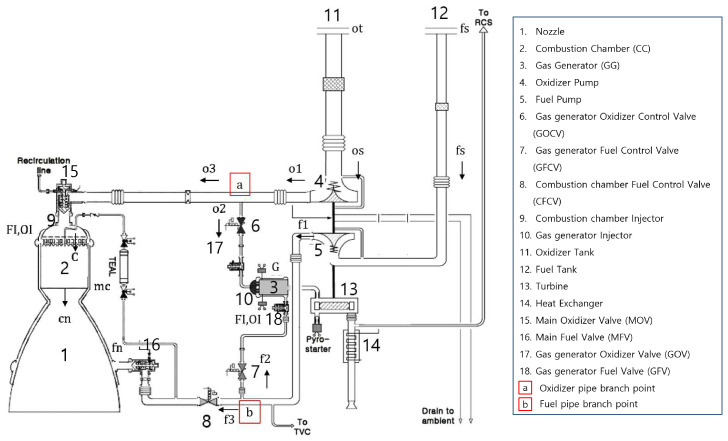
A schematic of a liquid-propellant rocket engine [32].

Using the MATLAB/Simulink environment, we constructed an open-cycle LPRE simulation program for dynamic simulation [32] for the open-cycle LPRE employed in this study, as shown in Figure 2. The mathematical model is described in more detail in Appendix A.

The open-cycle LPRE is analyzed differently depending on whether the operating valves are in a steady state or transient state [33]. During the startup process, one of the transient states, four main valves (Nos. 15, 16, 17, and 18 in Figure 2) affect the performance of the engine and cause it to operate at full thrust as swiftly as possible. Upon reaching the steady state, known as full thrust, the LPRE performance can be controlled using three control valves (Nos. 6, 7, and 8 in Figure 2). Finally, during the shutdown process, another transient state, the LPRE operation is terminated by the four main valves.

To ensure the accuracy of the simulation results, they were compared with experimental data from the steady and transient states. In the steady state (full thrust) of the simulation, with all valves and parameters fixed, a maximum error of 3.7% and a minimum error of 0% were observed [32]. However, during transient states such as the startup or shutdown procedures, four main valves were fully opened and closed at the appropriate time, respectively [16]. The comparison of transient states (startup and shutdown processes) is provided in Appendix B.

### 2.2. Fault Modeling

A fault is an unexpected change or an unpermitted deviation in system parameters from the standard no-fault condition, so a fault in a system may lead to system malfunction or mission failure [27]. This can be performed mathematically by combining the corresponding fault types; hence, we artificially injected each fault signal following the proven approach outlined in Refs. [15,20], which is verified with actual datasets. For the fault conditions in this study, turbopump faults and pipeline blockages, which are representative faults [15,20,27], are considered sudden faults:

(1) $$\theta \left(t\right)=\left\{\begin{array}{ccc} \theta_{0} & \mathrm{for} & t<t_{0}\\ \theta_{0}\left(1-f\right)≜\theta_{1} & \mathrm{for} & t_{0}\leq t \end{array}\right.$$

where θ is a parameter of the system, $\theta_{0}$ is the parameter in a normal condition, and $f=\frac{\Delta \theta }{\theta_{0}}$ represents the fault factor.

Even though Equation (1) describes fault development as a step function, the fault mathematical model can also be modeled by applying linear, quadratic, cubic, and exponential functions and changing the fault development time depending on the fault characteristics.

#### 2.2.1. Turbopump Fault

There are many factors for a turbopump fault, such as cavitation, rotor bearing faults, and turbine blade faults, which are difficult to implement individually by mathematical modeling. However, most faults in a pump or turbine induce a decrease in efficiency. Therefore, using Equation (1), we express a fault condition mathematically by injecting an efficiency decrease ($\theta =\eta_{t}$ for turbine or $\eta_{p}$ for pump in Equation (1)) considering an abruptly occurring ablation of turbine nozzle blades [15,20].

#### 2.2.2. Pipeline Fault

Pipeline faults are mostly leakages or hole blockages [15,20], but we only consider a pipeline blockage to be simple in this study by decreasing the pipe cross-sectional area (*A*). For this, using Equation (1), we express a blockage fault due to some impurities in propellants by injecting the area variation (θ=A in Equation (1)) into a part of the pipeline (CC oxidizer pipeline (o3) and CC fuel pipeline (f3) in Figure 2).

## 3. Particle Filter for Residual Generation

This section delineates the PF to generate the residuals for an FDD algorithm of an open-cycle LPRE. In previous research, Cha et al. [21] developed the FDD algorithm with the EKF/UKF. However, the linearization process in the EKF yields approximation errors in each prediction/update step, and it is difficult to globally approximate based on a small set of trial points in the UKF [29,30]. Consequently, these issues may lead to poor detection or high false alarm rates for the FDD method. The PF, a method based on Monte Carlo simulation principles for a nonlinear and non-Gaussian dynamic model, is appealing for its ability to adeptly handle various nonlinear distribution characteristics of measurement noise because it updates probability densities using a Bayesian approach [34,35].

The dynamic simulation program can be expressed using continuous state dynamics and a discrete measurement procedure as follows:

(2) $$\dot{x}\left(t\right)=f\left(x(t),u(t)\right)+w\left(t\right),$$

(3) $$z_{k}=h\left(x_{k}\right)+v_{k},$$

where $u_{k}\in R^{m}$, $x_{k}\in R^{n}$, and $z_{k}\in R^{l}$ are the input, state, and output variables, respectively, and $w\left(t\right)\in R^{n}$ and $v_{k}\in R^{l}$ are the state and measurement noises, respectively, assuming normal distributions with the covariances $Q\left(t\right)$ and $R_{k}$, respectively.

**Figure 3:**
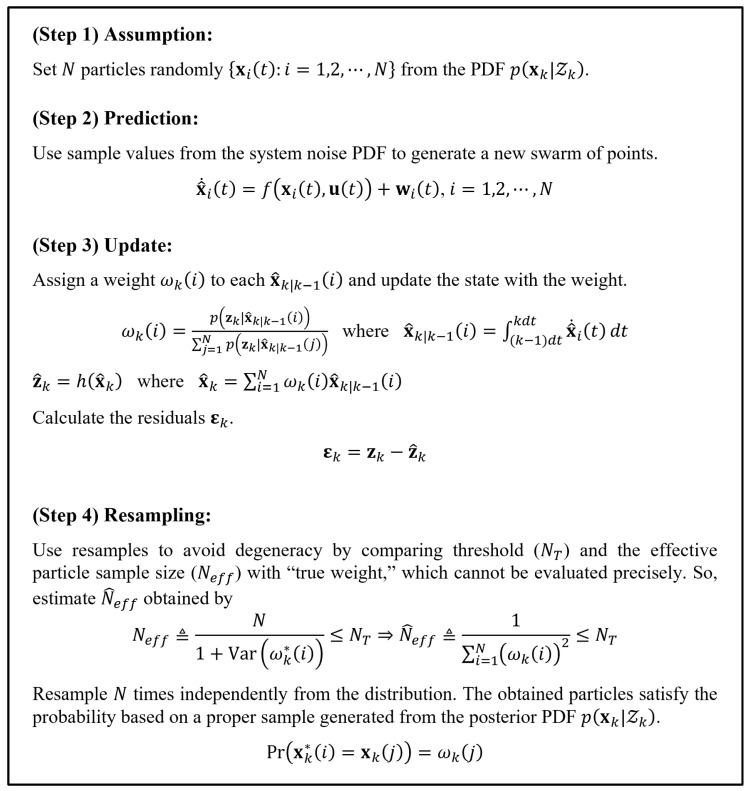
The four-step process for the particle filter [34].

For the nonlinear system, the PF was proposed to represent and recursively generate an approximation for the conditional probability density function (PDF), $p\left(x_{k}|Z_{k}\right)$, where $Z_{k}=\left\{z_{1},z_{2},\cdots ,z_{k}\right\}$. The fundamental concept of the PF is to obtain and portray the necessary PDF using particles in a swarm. The swarm can be regarded as the realization of random samples from the required PDF in each step. Thus, as the number of particles increases, they tend to approach the necessary PDF more closely [34,35]. Figure 3 illustrates the process of the PF algorithm. Then, we generate the residuals ($\epsilon_{k}$) using the system output variables ($z_{k}$) and estimated output variables (${\hat{z}}_{k}$) as follows:

(4) $$\epsilon_{k}=z_{k}-{\hat{z}}_{k}$$

## 4. Fault Detection and Diagnosis Using Residuals

Now, the CUSUM and MM methods use the residuals generated by the PF for the FDD process. As illustrated in Figure 1, the algorithm progresses through two blocks after residuals are generated by the PF: (1) the residual analysis block to detect faults and (2) the MM method block to diagnose faults.

### 4.1. CUSUM Algorithm for Fault Detection

**Figure 4:**
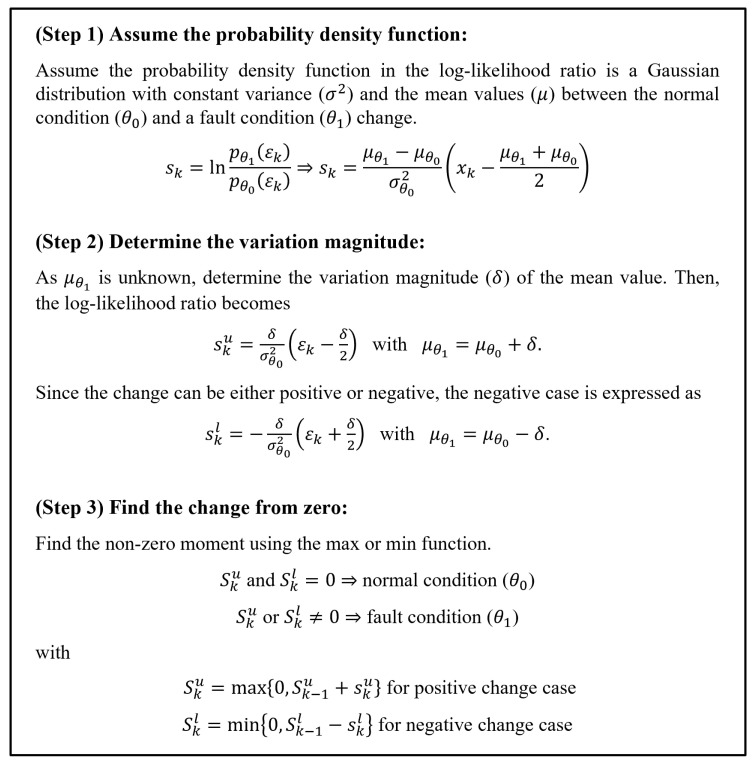
The three-step process of the CUSUM algorithm [37].

The residual analysis process determines whether a fault occurred through the residual-change-checking method, for example, by identifying whether a signal exceeds a threshold [36]. However, because the sensor measurements may contain anomalies due to various factors, such as noise, impulsive noise can be associated with the threshold being exceeded during the data acquisition process or by the sensor itself. Therefore, to overcome this problem, this study proposes a residual analysis method based on a modified CUSUM algorithm, widely used for fault detection [37]. As the mean value of the residuals generated by a filter shifts when a fault occurs, the algorithm can detect whether and when a fault occurs by checking a change in the mean values between the normal condition ($\theta_{0}$) and a fault condition ($\theta_{1}$) (see Equation (1)) [16]. Figure 4 presents the process of the CUSUM algorithm, through which we can detect a fault considering two cases by checking whether the CUSUM algorithm results ($S_{k}^{u}$ and $S_{k}^{l}$) are zero or not. Since the results are zero until the absolute value of each residual ($|\epsilon_{k}|$) exceeds δ/2, the variation magnitude (δ) in the process is a tuning parameter. Hence, the magnitude (δ) should be determined by considering the factor of safety (FS) of the engine. Generally, the FS depends on the components and engine types, but it is usually at least 1.1 (10% FS), especially for the combustion chamber because of combustion instability [38,39]. Therefore, here, we set the variation magnitude (δ) to 15% (δ/2=7.5%) of each output variable under the normal condition to identify a fault occurring before exceeding the 10% FS.

**Figure 5:**
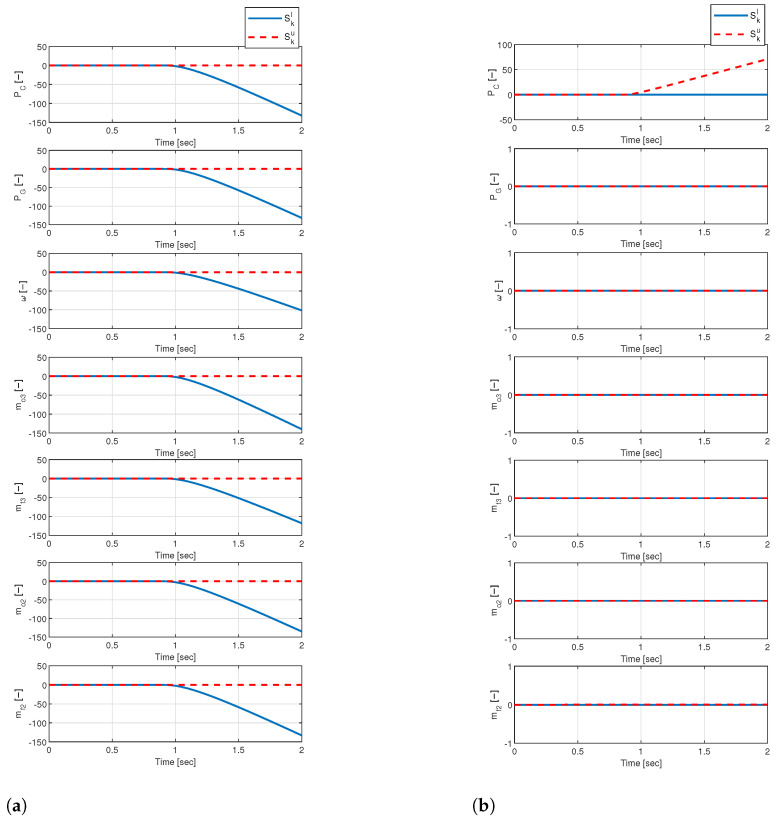
The results of the CUSUM algorithm (**a**) internal component fault results; (**b**) sensor fault results.

**Figure A2:**
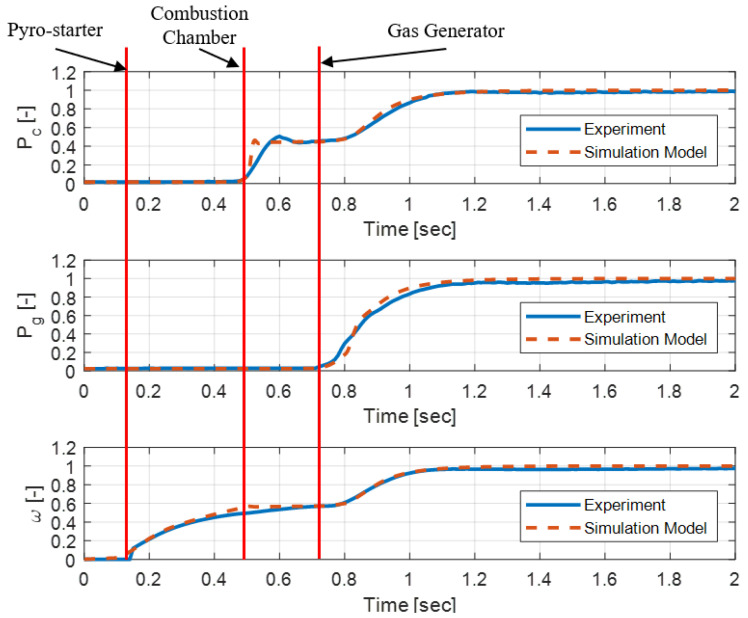
Comparison of the startup process between the simulation and experimental data. (**Top**) Combustion chamber pressure, (**Middle**) gas generator pressure, and (**Bottom**) turbopump rotational speed [16].

To see the algorithm performance, we applied the algorithm to two fault cases: a decreased turbine efficiency fault for internal faults and the CC pressure sensor positive bias fault for sensor/valve faults. For this, we artificially injected each fault at 0.9 s during the startup process (see Figure A2) and utilized the CUSUM algorithm using the residuals generated by the PF. Figure 5 shows the results of the CUSUM algorithm for a turbine efficiency fault (Figure 5a) and the CC pressure sensor bias fault (Figure 5b) using seven normalized state variables: $P_{c}$, $P_{g}$, ω, ${\dot{m}}_{o3}$, ${\dot{m}}_{f3}$, ${\dot{m}}_{o2}$, and ${\dot{m}}_{f2}$ (see Table 1). In the turbine efficiency fault case, the fault affects all engine components, so all the variables change. On the other hand, a sensor bias fault causes only the corresponding sensor output variables to change. Therefore, all results of the CUSUM algorithm in Figure 5a change in the negative case (blue line, $S_{k}^{l}$), while in Figure 5b, only the CC pressure ($P_{c}$) changes in the positive case (red line, $S_{k}^{u}$) and others do not. Through this process, we can see that the CUSUM algorithm can detect a fault by checking the change in the residuals. Furthermore, we can deduce from additional information that the CUSUM algorithm can distinguish between the internal component fault and the sensor fault from the number of changes in the results.

### 4.2. Multiple-Model Method for Fault Diagnosis

Once a fault is detected, the next step is to diagnose the fault, determining its location and severity. The MM method, consisting of multiple filters, is employed for fault diagnosis after the initial detection [16]. The decision mechanism is based on the residuals generated by a set of *N* filters. Each filter is designed based on different hypothesized models. If one of the hypothesized models is true, the residuals and covariance generated by the corresponding filter are small. Therefore, the hypothesized model that produces the smallest residual and covariance can be regarded as the true model [21]. This study assumes that a fault occurs in each of the five components for each hypothesized model, including the efficiency decreases among the turbine, fuel pump, and oxidizer pump, and two blockages in the fuel and oxidizer pipelines for the hypothesized models, and the artificially injected fault signature in the turbine efficiency at 0.9 s (see Figure A2) [16].

**Figure 6:**
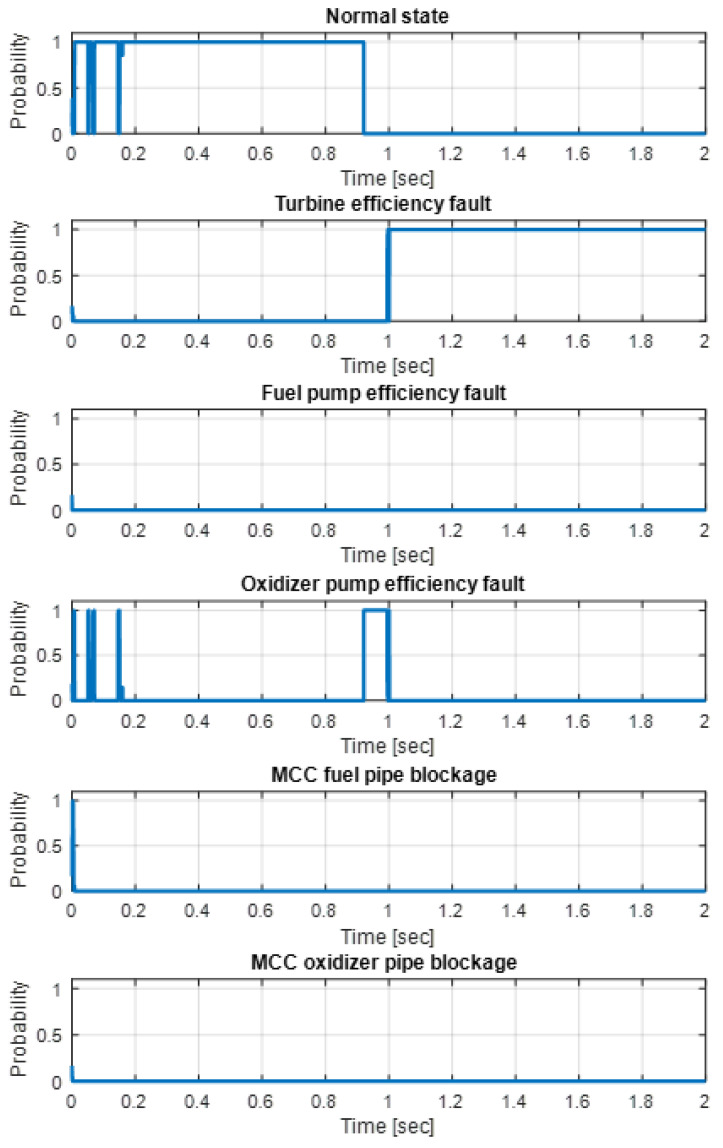
The results of the multiple-model method with the PF under the turbine efficiency fault [16].

The MM method results are depicted in Figure 6. Under the normal condition, the probability is mostly 1, and when the fault occurs, the probability changes to 0, and the turbine fault probability changes to 1. During this process, the probability of an oxidizer pump fault briefly reaches 1 due to the similarity of fault effects to those of turbine and oxidizer pump efficiency faults. Therefore, it can be confused at the beginning of the occurring fault, and the method can accurately diagnose the fault that eventually occurs.

## 5. PF-CUSUM-MM Application Results

**Figure 7:**
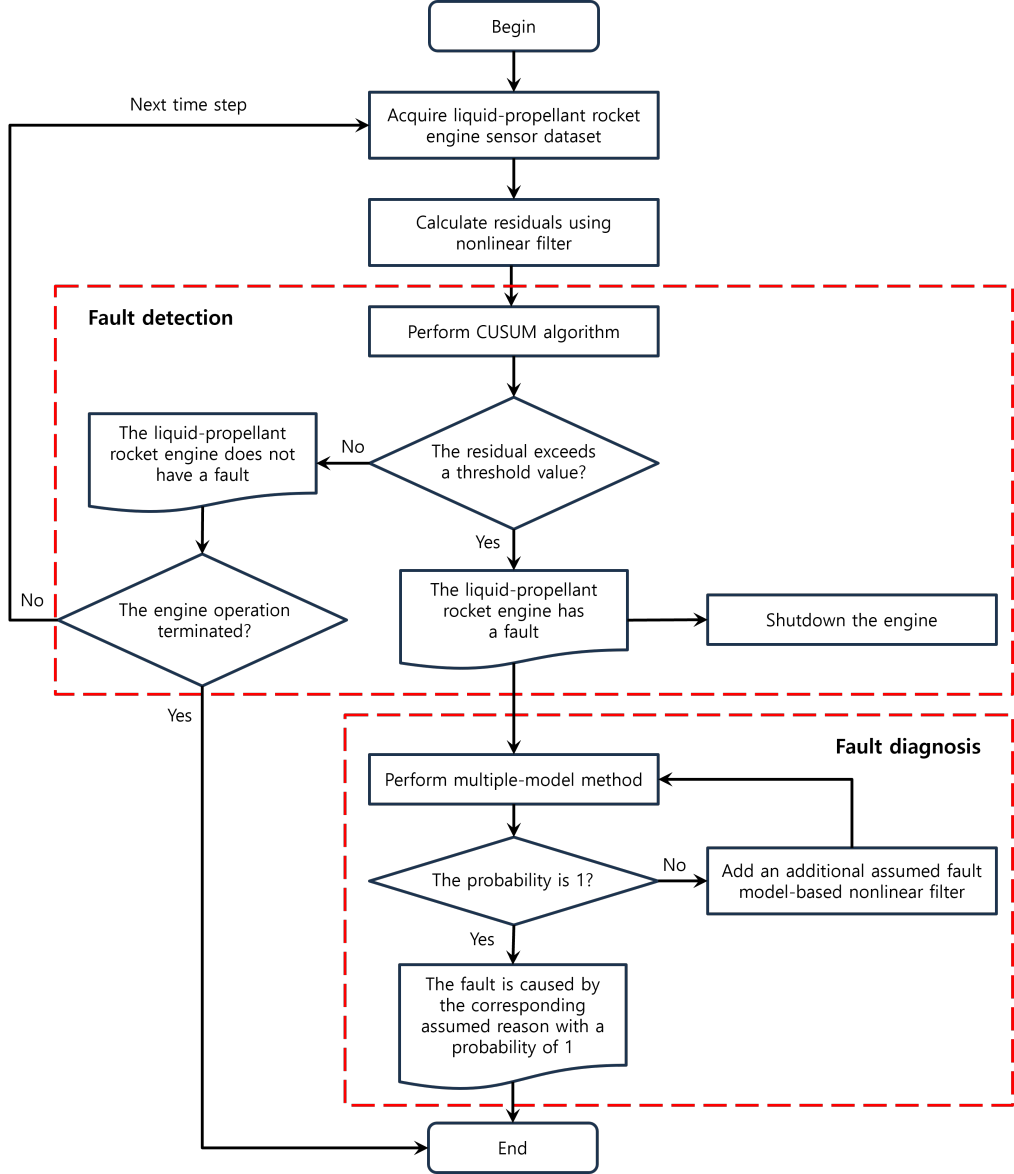
The flowchart of the proposed algorithm.

This section validates the feasibility of the proposed FDD algorithm. For this, we artificially injected a reasonable fault into the startup process using the minimum detectable fault analysis. Then, to evaluate the FDD algorithm, we used a Monte Carlo simulation in each process of the FDD algorithm and compared the performance with the previous results using the EKF/UKF. The process of the FDD algorithm is as follows: it first detects a fault using the residuals generated by a nonlinear filter, and then the fault diagnosis algorithm runs after the fault is detected. The overall flowchart of the proposed algorithm is shown in Figure 7.

### 5.1. Minimum Detectable Fault Analysis

To evaluate the performance of the FDD algorithm using an LPRE simulation with artificially injected faults, it is crucial to determine a reasonable initial fault magnitude for each fault model. Small fault sizes relative to the noise level can make detection and diagnosis challenging, potentially resulting in false alarms during the process [40]. Therefore, we analyzed the minimum detectable fault severity (MDFS) for five fault cases, each with four different fault magnitudes (5%, 10%, 15%, and 20%). This analysis compared the fault severity with the noise level, determined by the inverse of the signal-to-noise ratio (SNR) using Equation (5) (see Table 1), in scenarios involving efficiency decreases in the turbine, fuel pump, and oxidizer pump, as well as blockages in the CC oxidizer and CC fuel pipelines. However, since there are eleven output variables (n=11) and a fault affects each output variable differently, we used the MDFS of each fault by calculating the mean error rate value from the normal condition value of each output variable using Equation (6) and summarize the results in Table 2.

(5) $${\mathrm{SNR}}_{i}=\frac{E\left[s_{normal}^{i}\right]}{E\left[\sigma_{i}\right]},\;i=1,\ldots ,n,$$

(6) $$\mathrm{MDFS}=\frac{1}{n}\sum_{i=1}^{n}\frac{E\left[s_{fault}^{i}\right]-E\left[s_{normal}^{i}\right]}{E\left[s_{normal}^{i}\right]},$$

where $\sigma_{i}$ represents the standard deviation of the *i*-th output variable noise, and $s_{normal}^{i}$ and $s_{fault}^{i}$ are the *i*-th signal of the eleven output variables under normal and fault conditions, respectively.

All MDFSs for turbine and pump faults exceed the values listed in Table 1. This indicates that, even at the smallest fault magnitude (5%) for turbine or pump efficiency faults, the effects of the faults are greater than the noise effects. Therefore, the deviations can be identified as being caused by faults, rather than noise. In contrast, for the fuel pipeline blockage fault up to a magnitude of 5% and for the oxidizer pipeline blockage fault up to a magnitude of 15%, the MDFSs are smaller than the values listed in Table 1. Then, at those fault magnitudes, it becomes difficult to distinguish between deviations caused by faults and those caused by noise, which leads to an increase in false alarms. Therefore, this study uses 20% for each fault magnitude, where all MDFSs exceed all values listed in Table 1.

### 5.2. Performance Validation: A Comparison with the EKF and UKF

To confirm the algorithm performance, we compared the algorithms proposed in this study with those used in the previous study [21], which employed the other two nonlinear Kalman filters, focusing on a qualitative analysis. For this, a Monte Carlo simulation was conducted at each stage of the FDD algorithm, as illustrated in Figure 1. Subsequently, we compared the simulation results for the FDD algorithm based on each filter. In the Monte Carlo simulation process, we set two types of noise, Gaussian ($N\left(0,\sigma^{2}\right)$) and uniform ($U\left(-3\sigma ,3\sigma \right)$) distributions, and added them to the simulation data in each case.

**Figure 8:**
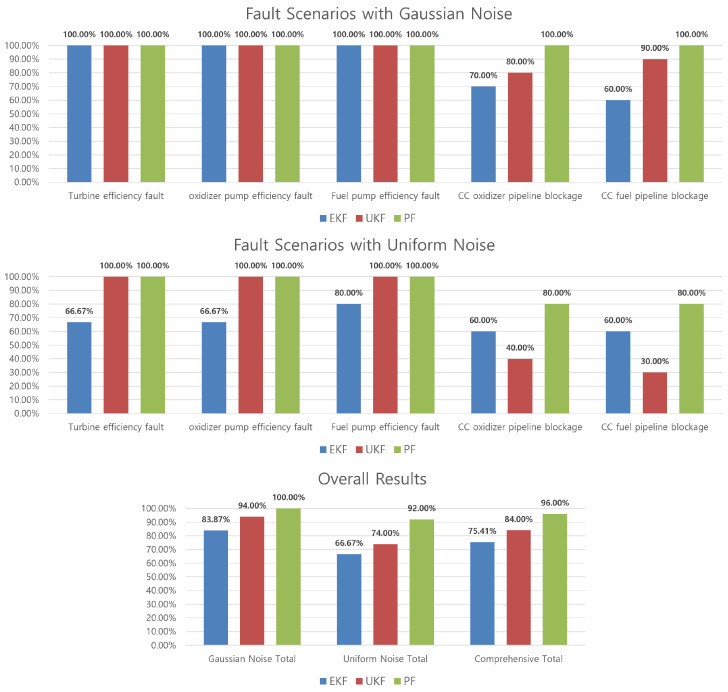
Monte Carlo simulation results of the CUSUM algorithm.

Figure 8 shows the comparison results of the CUSUM algorithm based on the EKF, UKF, and PF with Gaussian and uniform noises. The results of each detection probability show that the CUSUM algorithm, using the residuals from the PF, is better than the other nonlinear filters. The CUSUM algorithm using the UKF is better than the EKF, except for in some fault cases (in the pipeline blockage fault) with a uniform noise distribution. This could be because the UKF generates the residuals using a set of points obtained from the Gaussian distribution, whereas the EKF uses the linearization approach. Therefore, under noise distributions other than the Gaussian distribution, the algorithm based on the UKF may yield worse results compared to those with the EKF.

**Figure 9:**
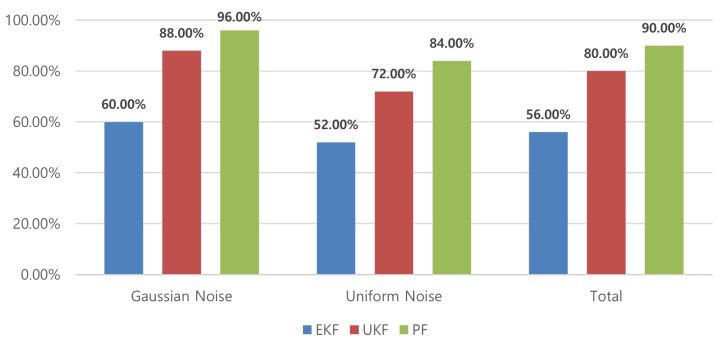
Monte Carlo simulation results of the multiple-model method.

Subsequently, a Monte Carlo simulation was conducted using the MM method to compare the performance in the diagnostic process by applying the residuals and error covariances generated by each filter. Figure 9 shows that the MM method, employing residuals and covariance derived from the PF, outperforms the other nonlinear filters (EKF/UKF).

According to these two results, the PF is superior to the other filters in the Monte Carlo simulation. In addition, since the algorithm with the PF has a computational cost and performance that depend on the number of particles, the FDD algorithm combined with the PF is expected to demonstrate greater effectiveness in posterior analysis, independent of computational complexity. For this analysis, the algorithm utilizes secured flight status and system condition datasets transmitted via telemetry devices during flight missions as the system output variables. Through this process, the cause of the fault can be determined and analyzed. Subsequently, this posterior analysis will facilitate failure analysis and enable learning from mistakes. It will also ensure the reliability of launch vehicles and the successful performance of Post-Mission Disposal (PMD) [26].

## 6. Conclusions

This study developed an FDD algorithm consisting of a PF, CUSUM algorithm, and MM approach for an open-cycle LPRE in the startup process using a nonlinear simulation verified using experimental data from both the steady and transient states. We then designed a PF to generate residuals and detected and diagnosed faults using a modified CUSUM algorithm and MM method. To assess the FDD algorithm, we numerically evaluated the performance of the CUSUM algorithm and the MM method in each FDD step. Subsequently, we compared the performance with that obtained in a previous study [21] using the Monte Carlo simulation under various fault conditions and noise distributions. In this study, the FDD algorithm based on the PF performed better than the FDD algorithm employing the other nonlinear filters, on average. Furthermore, considering the balance between computational resources and algorithmic performance, the FDD algorithm utilizing the PF can enhance effectiveness, particularly in posterior analysis tasks. The procedures used for other fault cases can be found in Cha’s study [16].

## Figures and Tables

**Table 1:** Inverse of signal-to-noise ratio.

Notation	Meaning	1/SNR [%]
$P_{C}$	Combustion chamber pressure	0.47
$P_{G}$	Gas generator pressure	0.51
${\dot{m}}_{o3}$	Combustion chamber oxidizer flow rate	0.48
${\dot{m}}_{f3}$	Combustion chamber fuel flow rate	0.57
ω	Turbopump rotational speed	0.40
${\dot{m}}_{OIC}$	Combustion chamber oxidizer injector flow rate	0.48
${\dot{m}}_{FIC}$	Combustion chamber fuel injector flow rate	0.57
${\dot{m}}_{OIG}$	Gas generator oxidizer injector flow rate	1.88
${\dot{m}}_{FIG}$	Gas generator fuel injector flow rate	2.29
${\dot{m}}_{o2}$	Gas generator oxidizer flow rate	1.88
${\dot{m}}_{f2}$	Gas generator fuel flow rate	2.29

**Table 2:** The minimum detectable fault severity of each fault type and magnitude [16].

Fault Type	Fault Injection Level [%]							
	5%		10%		15%		20%	
	Peak	Final	Peak	Final	Peak	Final	Peak	Final
Turbine efficiency	5.88	5.88	11.80	11.80	17.77	17.77	23.78	23.78
Oxidizer pump efficiency	3.11	3.11	6.39	6.39	9.86	9.86	13.54	13.54
Fuel pump efficiency	2.82	2.82	5.81	5.81	8.99	8.99	12.39	12.39
CC oxidizer pipeline blockage	0.48	0.39	1.03	0.84	1.66	1.35	2.39	1.94
CC fuel pipeline blockage	1.29	1.11	2.67	2.32	4.15	3.61	5.74	5.01

**Figure A1:**
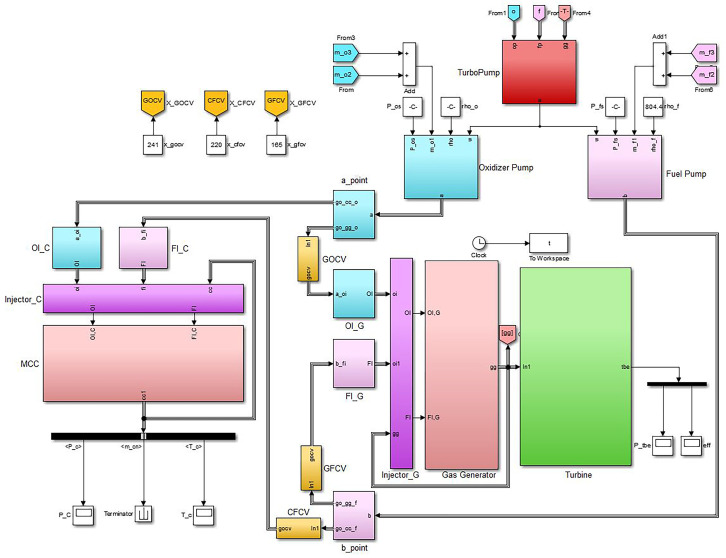
Simulation model of liquid-propellant rocket engine [32].

**Figure A3:**
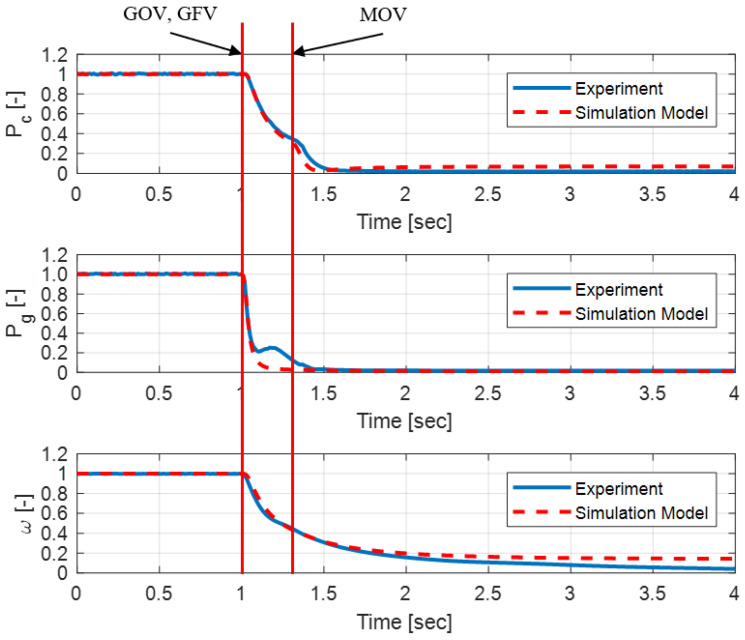
Comparison of the shutdown process between the simulation and experimental data. (**Top**) Combustion chamber pressure, (**Middle**) gas generator pressure, and (**Bottom**) turbopump rotational speed [16].

## Data Availability

Data are contained within the article.

## Abbreviations

The following abbreviations are used in this manuscript:

*A*	Cross-area of pipe
$I_{tp}$	Moment of inertia of turbopump rotor
*L*	Length of pipe
$P_{a}$	Oxidizer pipe branch point (a) pressure
$P_{b}$	Fuel pipe branch point (b) pressure
$P_{C}$	Combustion chamber pressure
$P_{fic}$	Combustion chamber fuel injector inlet pressure
$P_{fig}$	Gas generator fuel injector inlet pressure
$P_{G}$	Gas generator pressure
$P_{oic}$	Combustion chamber oxidizer injector inlet pressure
$P_{oig}$	Gas generator oxidizer injector inlet pressure
$R_{C}$	Combustion chamber gas constant
$R_{G}$	Gas generator gas constant
$T_{C}$	Combustion chamber temperature
$T_{G}$	Gas generator temperature
$V_{C}$	Combustion chamber volume
$V_{G}$	Gas generator volume
${\dot{m}}_{cn}$	Nozzle inlet flow rate
${\dot{m}}_{FIC}$	Combustion chamber fuel injector flow rate
${\dot{m}}_{FIG}$	Gas generator fuel injector flow rate
${\dot{m}}_{f2}$	Gas generator fuel flow rate
${\dot{m}}_{f3}$	Combustion chamber fuel flow rate
${\dot{m}}_{G}$	Gas generator outlet flow rate
${\dot{m}}_{OIC}$	Combustion chamber oxidizer injector flow rate
${\dot{m}}_{OIG}$	Gas generator oxidizer injector flow rate
${\dot{m}}_{o2}$	Gas generator oxidizer flow rate
${\dot{m}}_{o3}$	Combustion chamber oxidizer flow rate
${\dot{m}}_{G}$	Generated mass flow rate from solid propellant
$\epsilon_{fc}$	Amount of time delay in combustion fuel injector
$\epsilon_{fg}$	Amount of time delay in gas generator fuel injector
$\epsilon_{oc}$	Amount of time delay in combustion oxidizer injector
$\epsilon_{og}$	Amount of time delay in gas generator oxidizer injector
λ	Pressure loss coefficient
$\rho_{f}$	Fuel density
$\rho_{o}$	Oxidizer density
$\tau_{fp}$	Fuel pump torque
$\tau_{op}$	Oxidizer pump torque
$\tau_{tb}$	Turbine torque
ω	Angular velocity of turbopump

## Appendix A. Mathematical Model of the Liquid-Propellant Rocket Engine

The mathematical modeling of the LPRE used in this paper can be expressed in the form of differential and algebraic equations (DAEs). The differential equations are derived from seven governing equations, while the algebraic equations are derived from either empirical equations or the laws of thermal-fluid dynamics [33]. To streamline the LPRE simulation model, we utilized four governing equations, assuming a constant coefficient representation for heat transfer dynamics in the mass flow rate dynamics. Using this approach, we developed an open-cycle LPRE simulation program that employs 11 first-order ordinary differential equations and 37 algebraic equations [32]. The 11 nonlinear equations of motion derived from the governing equations are listed in Table A1. Additionally, the highest level of the simulation program in the MATLAB/Simulink environment is depicted in Figure A1.

**Table A1:** Dynamic model equations.

Number	Governing Equation	Mathematical Equation
1	Rotational dynamics	$I_{tp}\frac{d\omega }{dt}=\tau_{tb}-\tau_{op}-\tau_{fp}$
2	Pipe dynamics	$\left(\frac{L}{A}\right)\frac{d{\dot{m}}_{o3}}{dt}=P_{a}-P_{oic}-\left(\frac{\lambda }{2\rho_{o}A^{2}}\right){\dot{m}}_{o3}^{2}$
3		$\left(\frac{L}{A}\right)\frac{d{\dot{m}}_{f3}}{dt}=P_{b}-P_{fic}-\left(\frac{\lambda }{2\rho_{f}A^{2}}\right){\dot{m}}_{f3}^{2}$
4		$\left(\frac{L}{A}\right)\frac{d{\dot{m}}_{o2}}{dt}=P_{a}-P_{oig}-\left(\frac{\lambda }{2\rho_{o}A^{2}}\right){\dot{m}}_{o2}^{2}$
5		$\left(\frac{L}{A}\right)\frac{d{\dot{m}}_{f2}}{dt}=P_{b}-P_{fig}-\left(\frac{\lambda }{2\rho_{f}A^{2}}\right){\dot{m}}_{f2}^{2}$
6	Pressure dynamics	$\left(\frac{V_{c}}{R_{c}T_{c}}\right)\frac{dP_{C}}{dt}={\dot{m}}_{OIC}+{\dot{m}}_{FIC}-{\dot{m}}_{cn}$
7		$\left(\frac{V_{g}}{R_{g}T_{g}}\right)\frac{dP_{G}}{dt}={\dot{m}}_{OIG}+{\dot{m}}_{FIG}-{\dot{m}}_{G}$
8	Time-delay equation	$\frac{d{\dot{m}}_{OIC}}{dt}=\frac{1}{\epsilon_{oc}}\left({\dot{m}}_{o3}-{\dot{m}}_{OIC}\right)$
9		$\frac{d{\dot{m}}_{FIC}}{dt}=\frac{1}{\epsilon_{fc}}\left({\dot{m}}_{f3}-{\dot{m}}_{FIC}\right)$
10		$\frac{d{\dot{m}}_{OIG}}{dt}=\frac{1}{\epsilon_{og}}\left({\dot{m}}_{o2}-{\dot{m}}_{OIG}\right)$
11		$\frac{d{\dot{m}}_{FIG}}{dt}=\frac{1}{\epsilon_{fg}}\left({\dot{m}}_{f2}-{\dot{m}}_{FIG}\right)$

## Appendix B. Verification of Dynamic Simulation

We verified the simulation results by comparing the startup and shutdown processes with the normalized simulation and experimental data of three state variables: $P_{c}$, $P_{g}$, and ω (see Table 1). In Figure A2, the startup process is delineated, progressing sequentially through the pyro-starter, CC ignition, and GG ignition stages. In the startup process, the pyro-starter first rotates the turbopump, and then ignition starts combustion in the CC. Finally, combustion begins in GG, and the turbopump accelerates until the full thrust is achieved.

In contrast, Figure A3 shows the shutdown process, delineating a sequential pattern. First, the GG oxidizer valve (GOV) and GG fuel valve (GFV) close to reduce the turbopump rotation speed and the propellant flow to the CC. Afterward, when the propellant flow to the CC is sufficiently reduced, the main oxidizer valve (MOV) closes, terminating engine operation.
